# Design and Construction of the Optical Bench Interferometer for the Taiji Program

**DOI:** 10.3390/s23229141

**Published:** 2023-11-13

**Authors:** Wei Tao, Xiaoqin Deng, Yuqing Diao, Ruihong Gao, Keqi Qi, Shaoxin Wang, Ziren Luo, Wei Sha, Heshan Liu

**Affiliations:** 1 Changchun Institute of Optics, Fine Mechanics and Physics, Chinese Academy of Sciences, Changchun 130033, China; taowei19@mails.ucas.edu.cn (W.T.); dengxiaoqin19@mails.ucas.edu.cn (X.D.); diaoyuqing21@mails.ucas.edu.cn (Y.D.); 2 University of Chinese Academy of Sciences, Beijing 100049, China; 3 Key Laboratory of Optical System Advanced Manufacturing Technology, Chinese Academy of Sciences, Changchun 130033, China; 4 National Microgravity Laboratory (NML), Institute of Mechanics, Chinese Academy of Sciences, Beijing 100190, China; gaoruihong@ucas.ac.cn (R.G.); qikeqi1985@126.com (K.Q.); wangshaoxin@imech.ac.cn (S.W.); luoziren@imech.ac.cn (Z.L.)

**Keywords:** optical bench interferometer, hydrogen–oxygen catalytic bonding, space gravitational wave detection, Taiji program

## Abstract

A kind of full-function two-sided optical bench interferometer (OBI) is designed to meet the practical requirements of the Taiji Program for space gravitational wave detection. The main optical paths are arranged on the A-side for transmission and interference, and other optical paths and electronic devices are placed on the B-side. According to the design scheme, we successfully constructed two OBIs by using hydrogen–oxygen catalytic stress-free bonding technology. When the OBI is installed and adjusted, the position and Angle error of the interference beam are controlled within 30 μm and 50 μrad through the self-designed precision mechanical clamping mechanism and beam position measuring device. The built OBI was placed on the vibration isolation platform in the vacuum tank for the stability test. The test results show that the noise of the OBI is less than 10 pm/√Hz in the frequency band of 0.1 Hz to 1 Hz, which meets the noise budget requirements of the Taiji Pathfinder in the middle- and high-frequency band.

## 1. Introduction

Since the Laser Interferometer Gravitational-wave Observatory (LIGO) announced the detection of gravitational waves in 2016 [1,2], which verified Einstein’s prediction of gravitational waves more than a century ago, the research and detection of gravitational waves have drawn a lot of attention. China announced the Taiji Project, a space gravitational wave detection project, in 2016. Unlike ground-based gravitational wave detection, which has difficulty detecting gravitational waves with frequencies below 1 Hz, Taiji detects gravitational waves in the 0.1 mHz to 1 Hz band with a center frequency of 3 mHz. Taiji has the advantages of lower detection frequency, more abundant gravitational wave sources, no interference from earthquakes and human activities, a long detector baseline, and high sensitivity.

**Figure 1:**
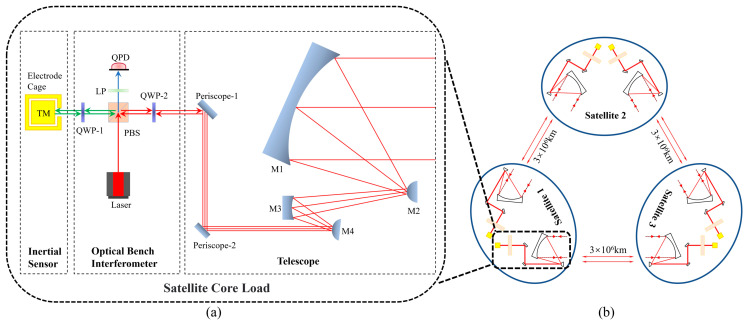
Structure diagram of core load composition in Taiji single-star and three-star constellation. (**a**) The core load of the satellite mainly includes the off-axis quad-mirror telescope subsystem, which receives and transmits lasers to build inter-satellite interference links; the optical measurement subsystem, which interferes with the local laser and the laser from a remote satellite; and the inertial sensor subsystem, which is used for satellite attitude control and sensing gravitational waves. (**b**) An equilateral triangular constellation of three identical satellites, 3 × 10^6^ km apart, each consisting of two sets of core loads.

Taiji plans to build an equilateral triangular three-star constellation in a heliocentric orbit about 20 degrees ahead of Earth and 1 astronomical unit from the Sun [3], as shown in Figure 1b, with satellites 3 million kilometers apart, using the principle of laser heterodyne interferometry to detect gravitational waves. LISA is another prestigious space gravitational wave detection project also made up of three satellites 2.5 million kilometers apart. Its orbit is similar to Taiji’s and lies about 20 degrees behind Earth on the other side. As the world’s first proposed space gravitational wave detection program, LISA’s technology has been highly mature and ahead of other space gravitational wave detection projects [4,5,6]. When gravitational waves pass through the constellation, spacetime will be distorted, resulting in changes in the distance between the satellites at the two ends of the interference arm, and those changes will be measured by the laser interferometer system to interpret the passing gravitational wave signal [3].

As shown in Figure 1a, the optical measurement subsystem is one of the core loads of the satellite, and the optical bench interferometer (OBI) is one of its main components. To detect gravitational waves, the optical path noise of Taiji must be less than 10 pm/√Hz in the band greater than 3 mHz [3], and the noise budget assigned to the OBI is 1 pm/√Hz. For the Taiji Pathfinder, the feasibility verification of various key technologies is the main goal, so the index is slightly reduced, and the optical path noise requirement for the OBI is less than 10 pm/√Hz.

To achieve this goal, during the study of the OBI prototype, the team researchers have made efforts to reduce the OBI noise from the aspects of phasemeter noise, laser beam direction control, Differential Wave-front Sensing (DWS) technology, laser phase locking, and optimization of the experimental environment [7]. However, these OBIs are built with invar or aluminum alloy as the substrate. Although the noise of the OBI has been reduced as much as possible, due to the influence of stress birefringence and elastic optical effect, the coefficient of thermal expansion between the glass optical element and the metal structure is very different. When the temperature changes, the stress will be generated, resulting in large and unstable optical path noise. The optical path noise of the OBI cannot be less than 10 pm/√Hz, which is not up to the requirements of Taiji Pathfinder.

To solve the problem of excessive optical path noise of the OBI, we successfully used hydrogen–oxygen catalytic bonding (HCB) technology to build two OBIs. HCB is a stress-free catalytic bonding technique that uses a covalent bond formed by a chemical reaction between a hydroxide solution and the surfaces of two silica (or other metal oxides) to form a strong connection [8]. This technology has already been used in LISA [9], another space gravitational wave detection project led by the European Space Agency, and it has been successfully used to build the ultra-stable OBI on the LISA Pathfinder technology verification satellite [10]. The maximum acceleration noise is 0.54 × 10^−15^ g/√Hz, and the range noise is 34.8 pm/√Hz, which is better than the expected target [6,11].

This paper introduces a full-function two-sided OBI designed for Taiji Pathfinder in detail. Different from the single-side optical path design of the LISA pathfinder OBI and only partial functions, the Taiji OBI is designed with optical paths on both the front and back of the substrate. The main interference optical path and other passive optical components are arranged on the A-side of the OBI, and the thermal electronic components, such as detectors and capture cameras, are arranged on the B-side. This design effectively isolates the influence of the heat devices on the optical path and reduces the noise caused by the stray light reflected by the optical elements into the detectors. The Taiji OBI includes not only scientific interferometers for interplanetary interferometry, reference interferometers, and test mass interferometers, but also interplanetary capture and sighting optical paths, pointing ahead optical paths, and backlink optical paths. With complete functions and a complex optical path layout, it realizes multi-optical multiplexing of the single optical element, which is close to the final form of the Taiji spaceborne OBI. It can be used to carry out more comprehensive inter-satellite link construction experiments in the ground laboratory, simulate the Taiji inter-satellite interferometry, verify the feasibility of relevant technologies, and provide a basis for the optimization of relevant parameters.

A precision mechanical device for clamping aligned optics was designed and constructed, with a step displacement resolution better than 60 nm. A beam position monitoring instrument was constructed with a position measurement resolution of 1 μm and an Angle measurement resolution of better than 10 μrad. The position and Angle deviation of the interference light path on the OBI are successfully controlled within 30 μm and 50 μrad. The OBI is constructed by using HCB stress-free bonding technology. Two OBIs are constructed by using Zerodur^®^ as the baseplate and bonding the fused silica glass optical elements on it.

Finally, a vacuum environment stability test was carried out to test the optical path stability and technical feasibility of the HCB. The test results show that the position noise of the OBI is less than 100 pm/√Hz in the band of 3 mHz to 0.1 Hz, less than 10 pm/√Hz in the band of 0.1 Hz to 1 Hz, and reaches 3 pm/√Hz at 1 Hz. The HCB technology has been successfully applied to the Taiji ultra-stable OBI, and the position noise of the OBI can meet the noise budget requirements of Taiji Pathfinder in the middle- and high-frequency band.

## 2. The Design of the OBI

The primary requirement for the OBI is that its optical path layout should be able to achieve all of the detection functions of the Taiji mission (including displacement noise measurement, communication ranging, inter-satellite acquisition and tracking, clock noise cancellation, etc.). Second, the OBI should achieve high stability, avoid the impact of thermal expansion and contraction of optical components on optical path measurement, and effectively reduce the noise of the OBI. Third, the OBI should also carry out a reasonable layout of the optical path to improve the space utilization of the platform. Finally, the OBI should have a solid and reliable connection to withstand the vibration and shock during the launch of the rocket.

**Figure 2:**
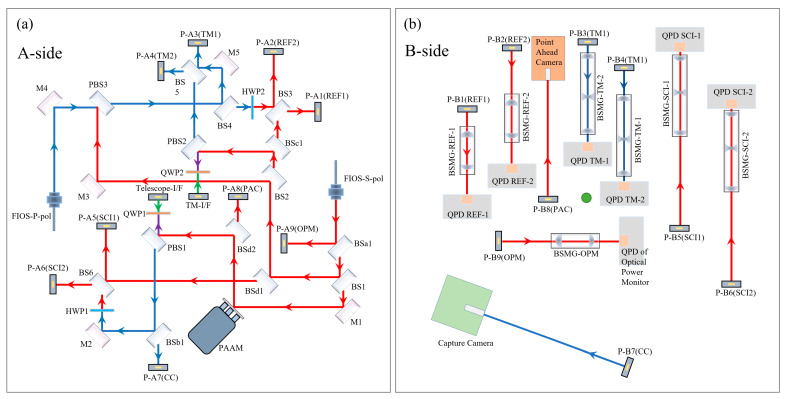
The OBI optical path, (**a**) the A-side, (**b**) the B-side. After frequency shift by AOMs, the laser is imported to the OBI through FIOSs. The different polarization states of the laser are distinguished by color. Blue indicates linearly polarized light parallel to the OBI plane; red indicates linearly polarized light perpendicular to the OBI plane; green indicates that linearly polarized light becomes elliptically polarized light after QWP; and purple indicates that the laser path represented by blue and red coincides. The blue and red lasers are converted to each other after HWP.

The complete optical path layout of the Taiji OBI is shown in Figure 2. The main optical elements are arranged on side A, and all heating elements are arranged on side B. Side A faces the Telescope (Figure 1a), and the reflector Telescope-I/F is the optical path transmission interface between the OBI and the telescope. The B-side faces the inertial sensing system, and the mirror TM-I/F is the optical path transmission interface between the OBI and the test mass (TM). See Table 1 for a detailed description of each optical element in the figure. The A-side contains three interferometers (the reference interferometer, the TM interferometer, and the scientific interferometer) and three auxiliary optical paths (Intersatellite Capture, Point Ahead Angle, and Backlink). The three interferometers carry out optical path transmission and complete interference on the A-side, and they are reflected by the periscopes to the B-side, where they are received and detected by the Quadrant Photodiodes (QPDs). This design can isolate the influence of heat components and the electromagnetic environment on the optical path signal and reduce the noise caused by stray light reflected by optical components into the detectors. The double-sided multiplexing of the OBI baseplate improves the space utilization rate, effectively reduces the size and weight of the OBI, and is conducive to future rocket launches.

**Figure 3:**
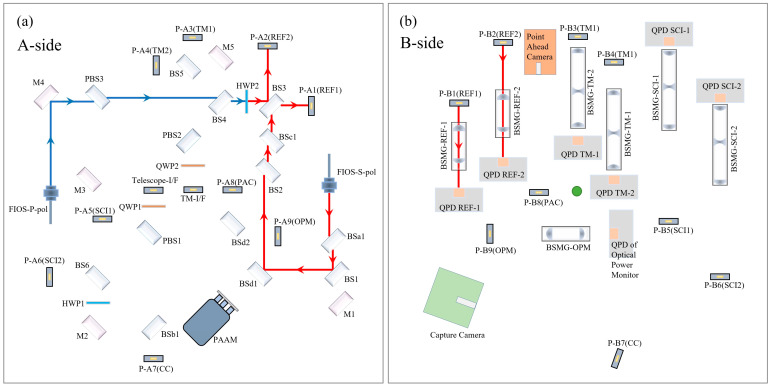
The reference interferometer optical path, (**a**) the A-side, (**b**) the B-side. The optical elements in the optical path remain stable, and neither of the two optical paths of this interferometer will change.

The reference interferometer is static. Due to the irreducible noise in the process of optical fiber transmission and connection, the reference interferometer can provide this part of the background noise for the TM interferometer and the scientific interferometer, which can be used to suppress the common mode noise between the interferometers and improve the measurement accuracy. The optical transmission path of the reference interferometer is shown in Figure 3. On the A-side, the P-polarization laser emitted by FIOS-P-pol and the S-polarization laser emitted by FIOS-S-pol come from the same laser. Their polarization directions are perpendicular to each other, and the difference frequency adjustment is performed by AOMs before entering the OBI. The P-polarization laser (blue) emitted from FIOS-P-pol is converted into S light at the HWP2, and the S-polarization laser from FIOS-S-pol interferes at BS3. The interference beam is equally divided into two parts and reflected by the periscope P-A1 (REF1) and P-A2(REF2) to the B-side. On the B-side, the interference beam with a diameter of 3.5 mm passes through the beam shrinking mirror group BMSG-REF for a beam reduction to 1 mm, and then it is received by the QPD REF with a diameter of 1.2 mm.

**Figure 4:**
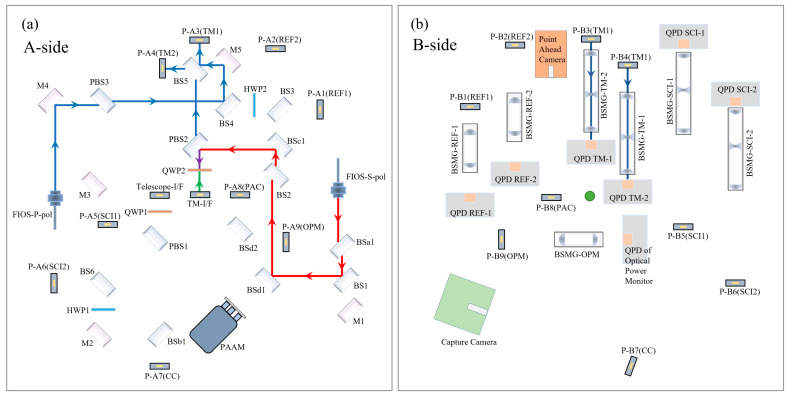
Optical transmission path of TM interferometer, (**a**) the A-side, (**b**) the B-side. The blue laser is the reference beam, and the red laser is the measurement beam. The red laser leaves the OBI from TM-I/F, turns back after reflection from the TM surface, and interferes with blue light at BS5.

The TM interferometer has two main functions. The first is to measure the displacement and Angle change of the TM relative to the OBI before detecting the scientific signal and to provide data for the satellite to adjust the attitude so that the TM in the electrode cage can maintain static equilibrium with no drag motion state. The second is to detect the scientific signal by monitoring the displacement change of TM in the scientific detection stage. The optical transmission path of the TM interferometer is shown in Figure 4. On the A-side, the S-polarization laser (red) emitted from FIOS-S-pol is reflected by the optical path interface TM-I/F to the TM opposite the B-side. After reflection from the TM surface, the original path returns and interferes with the P-polarization laser (blue) from FIOS-P-pol at BS5. The interference beam is equally divided into two parts, reflected by the periscope P-A3 and P-A4 to the B-side, and received by the detectors QPD TM.

**Figure 5:**
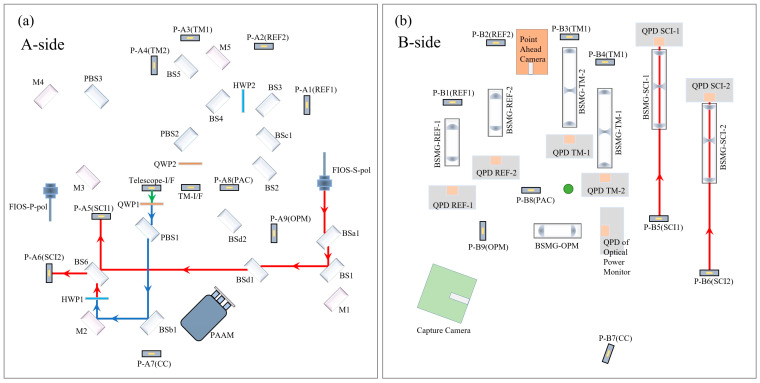
Optical transmission path of scientific interferometer, (**a**) the A-side, (**b**) the B-side. The red laser is the reference beam, and the blue laser is the measurement beam. The telescope will receive the laser emitted by another satellite and then import it into the OBI through the Telescope-I/F.

The scientific interferometer is formed by the laser emitted by the remote satellite and the local laser, which is used to measure the distance change information of the OBIs between the two satellites to obtain the gravitational wave signal. The laser transmission path of the scientific interferometer is shown in Figure 5. The laser emitted by the remote satellite is received by the Telescope and then imported into the OBI through the Telescope-I/F, where the exit pupil diameter of the telescope is 3.5 mm, and it interferes with the local laser (red) at BS6. The beam is reflected by the periscope P-A5 and P-A6 to the B-side. On the B-side, the beam passes through the BMSG-TM for a beam reduction, and it is then received by the detectors QPD SCI.

**Figure 6:**
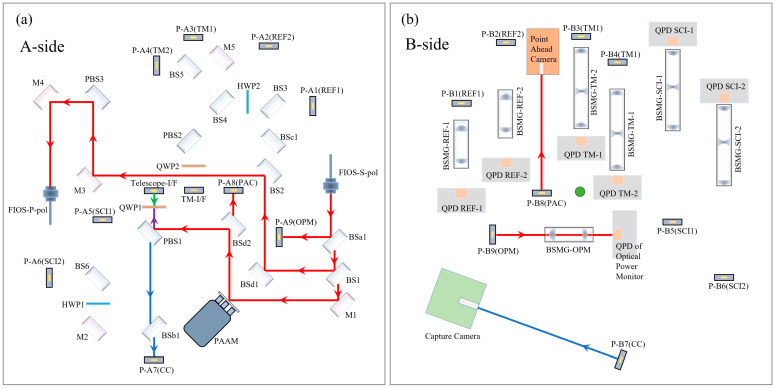
The auxiliary optical path, (**a**) the A-side, (**b**) the B-side. The Capture optical path starts from Telescope-I/F and ends with the capture camera. In the Point Ahead Angle optical path, the Point Ahead Camera is used to monitor the direction of the optical axis, and the PAAM is used to adjust the direction. The Backlink optical path starts from FIOS-S-pol and ends at FIOS-P-pol.

There are three auxiliary optical paths on the OBI, as shown in Figure 6. One is the Capture optical path (blue). The laser emitted by the remote satellite is received by the telescope and transmitted to the B-side of the OBI for reception by the Capture Camera. Its function is to capture the laser from the remote satellite and extract the information carried by the laser when constructing the inter-satellite interference link. The second is the Point Ahead Angle optical path. The PAAM is used to adjust the direction of the local emission laser optical axis to compensate for the advanced Angle caused by the relative motion between satellites. The Point Ahead Camera is used to monitor the difference between the emission laser optical axis and the theoretical optical axis. The third is the Backlink optical path, which is used to import the laser on one OBI into another OBI in the same satellite through the fiber, thus unifying the local laser signals on the two OBIs.

## 3. The Construction of the OBI

**Figure 7:**
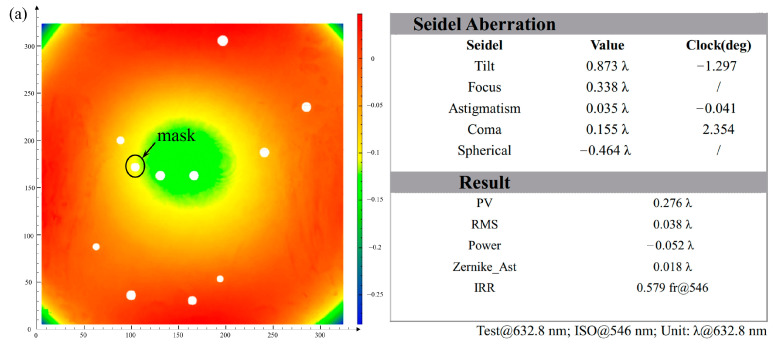

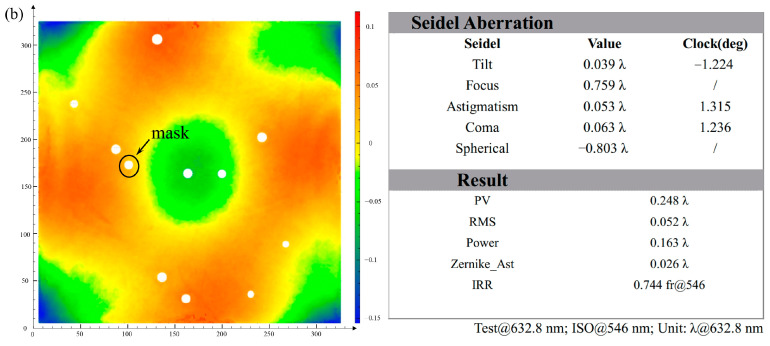
Surface profile test report of the Zerodur^®^ baseplate. (**a**) The A-side test result. (**b**) The B-side test result. Ten holes were processed on the baseplate, and the machining surface profiles of the two surfaces were measured with the Fizeau interferometer. Because there was a small contamination point on the reference surface of the measurement interferometer, we made a small “mask” at the point, so a blank was left at the same position on the measure results of the two surfaces, which did not affect the measurement results.

We built two OBIs for Taiji ground experiments. The OBI baseplate is a piece of Zerodur^®^ (Schott, Shanghai, China) that is 320 mm long, 320 mm wide, and 70 mm thick, with a mass of 18 kg. The Zerodur^®^ is a zero-expansion material, and its coefficient of thermal expansion (CTE) is less than 10^−7^/K, which can significantly reduce the thermal noise caused by temperature fluctuations. The thickness of 70 mm ensures that the OBI has a high enough stiffness without deformation. According to the requirements of the HCB process, the two surfaces for HCB need to be processed to the RMS value less than λ/10, λ = 632.5 nm to ensure successful and firm bonding [12]. Figure 7 shows the processing test report of one of the Zerodur^®^ baseplates. The PV value of the A-side is 0.276λ (λ = 632.8 nm), and the RMS value is 0.038λ. The PV value of the B-side is 0.248λ, and the RMS value is 0.052λ. All of these values can meet the requirements of the HCB process. Ten light-through holes with a diameter of 8 mm were machined into the Zerodur^®^ baseplate for transmitting the laser from A-side to B-side through the periscope.

The core optical components of the OBI are made of fused silica, which are bonded on a Zerodur^®^ baseplate using HCB technology. The brand of fused silica is Nikon NIFS-S (the CTE is 0.58 × 10^−6^/K, the refractive index is 1.45, and the temperature refractive index coefficient is 9.6 × 10^−6^/K), with good light transmission and optical uniformity. These fused silica blocks are processed into a uniform shape that is 20 mm long, 10 mm wide, and 35 mm high. They are finely polished and plated with dielectric films with different optical properties as required, as shown in Table 1. In addition, the verticality between the bottom plane and the optical plane is processed to less than 8 μrad (1.5 arcseconds), and the RMS value is less than λ/10, which can better ensure that the pitch angle of the optical path relative to the baseplate plane is small enough when the optical paths are built by the fused silica elements (less than 15 μrad).

### 3.1. HCB

A variety of solutions were considered for the connection of the Zerodur^®^ baseplate with the fused silica optical elements. The first was the use of epoxy resin glue for bonding; this technology is widely used in the field of aerospace optics with low cost, easy operation, strong connection, and other advantages. However, in the field of gravitational wave detection, the use of glue has obvious disadvantages. Epoxy resin adhesive requires a bonding layer thickness of at least tens of microns to ensure a solid and reliable bonding. However, the bonding layer of this thickness stores a certain amount of energy and has high internal stress, which will change the refractive index of fused silica and cause stress birefringence, which will bring huge and unpredictable optical path-length noise and drown out weak scientific signals [13]. In addition, the adhesive layer has poor thermal conductivity, the epoxy resin glue itself is volatile, and other shortcomings will bring thermal noise, which cannot meet the strict requirements of gravitational wave detection for noise, so the use of epoxy resin was excluded. Other schemes were also ruled out due to difficulties in meeting the low noise requirements. We finally chose the technology of HCB.

The HCB technology was first applied to Gravity Probe B in the space field [14], with which scientists combined dozens of silica parts into an all-glass telescope. This technology has also been successfully applied in the OBI of the LISA Pathfinder satellite and the tilt-to-length (TTL) optical bench, effectively solving the problem of optical path-length noise caused by optical elements stress and thermal noise, reducing OBI optical path-length noise to a very low level, and meeting the requirements of LISA for the detection of scientific targets [9,15,16].

The HCB technique involves placing a small amount of hydroxide solution (usually sodium hydroxide, potassium hydroxide, or sodium silicate) between glass and metal oxide or silicon carbide and using the chemical reaction between the hydroxide ion and the oxide to create a new material binding layer between the two bonded objects, leaving a new covalent bond grid connecting layer. This bonding layer is different from the physical bonding as it grows from between the two objects, it has more similar properties to the two bonds (such as CTE), and the bonding stress is lower. It is very suitable for application scenarios requiring low stress and high intensity, and it has been successfully applied in many fields (such as LIGO).

Its principle and chemical reaction process can be divided into three processes: hydration (and etching), polymerization, and dehydration [17,18].

Hydration and etching: The $OH^{-}$ ions in the solution act as a catalyst to corrode the silicon dioxide surface in contact with the solution, thus releasing silicate ions from the silicon dioxide surface:

(1) $$SiO_{2}+OH^{-}+2H_{2}O\overset{}{\to }Si(OH{)}_{5}^{-}$$

Polymerization: The total number of active $OH^{-}$ ions in the solution is reduced, so the pH of the bonded solution is reduced. Once the pH of the bonded solution drops below 11, the silicate ions dissociate to form $Si(OH{)}_{4}$:

(2) $$Si(OH{)}_{5}^{-}\overset{}{\to }Si(OH{)}_{4}+OH^{-}$$

$Si(OH{)}_{4}$ molecules can bind to each other and polymerize to form siloxane chains and water:

(3) $$2Si(OH{)}_{4}\overset{}{\to }(HO{)}_{3}SiOSi(OH{)}_{3}+H_{2}O$$

Dehydration: Water migrates or evaporates in it. During this period, the thickness of the bond layer decreases and the strength increases. At room temperature, after adding silicate solution between the two bonded surfaces, the chemical reaction can be completed within 1 min and it has a certain strength; the strength reaches the maximum after standing for a month.

**Figure 8:**
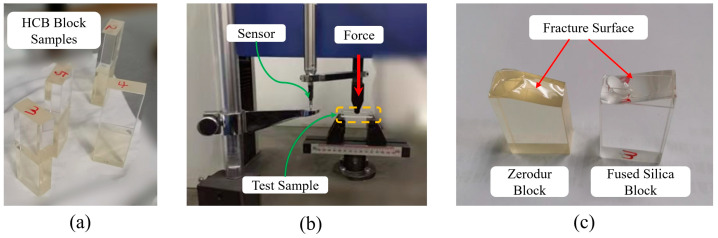
Strength test of HCB. (**a**) The Zerodur^®^ glass block and the fused silica glass block that had been bonded through HCB, the number on the block is the number of the sample, (**b**) the bonding strength test in progress, (**c**) the fracture section after the test.

Before the construction of the OBI, we carried out a series of tests on the strength of the HCB, as shown in Figure 8. The test results show that the bending strength is 35 ± 3 Mpa, the shear strength is 15 ± 1 Mpa, and the bonding strength meets the requirements. The glass blocks used in the test were machined into cuboid shapes with a height of 30 mm. The bonding interface is 20 mm × 10 mm and polished to an RMS value of less than λ/10, as required. The volumetric ratio of sodium silicate to water used is 1:6, and the amount of solution used is 0.4 μL/cm^2^.

### 3.2. Precision Clamping Mechanism

**Figure 9:**
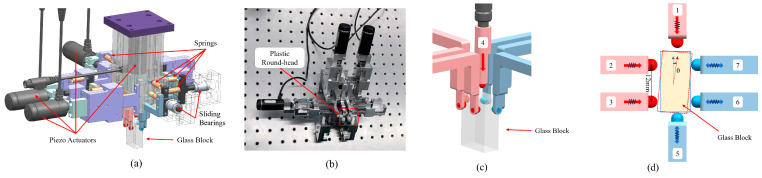
Precision clamping mechanism. (**a**) Three-dimensional model of the mechanism. (**b**) Physical object. (**c**) Local axial side diagram when the glass block is clamped. (**d**) Schematic diagram when the glass block is rotated.

A precise glass element clamping mechanism was designed and constructed for the precise installation and adjustment of the OBI. As shown in Figure 9, the clamping mechanism has seven fingers, six of which can clamp the glass block from the four facades, and one of which exerts force on the glass block from the top to make it move downward. Four of the seven fingers in red (numbers 1–4) are active fingers powered by piezo actuators (type: PZA12) from Newport^®^, which have a step displacement accuracy of 30 nm. The other three fingers act as passive fingers, and the six horizontal fingers all have lightweight springs as holding forces. Under the action of the clamping mechanism, the position movement and angular rotation step displacement of the glass block are 30 nm, 5 μrad in theory, but about 60 nm, 10 μrad in practice.

All of the places of the clamping mechanism in contact with the glass are made of plastic round-head screws to avoid scratching the glass. The clamping mechanism is installed at the end of the six-axis robot arm (from KUKA Company, type: KR10 R1100 WP), the repetitive positioning accuracy of the robot arm is 0.03 mm, and the maximum load is 10 kg. In the process of installation and adjustment, the robot arm first moves to the position near where the glass block will be bonded, and then the precision clamping mechanism at the end of the robot arm makes fine adjustments until the glass block reaches the predetermined position.

### 3.3. Beam Position Measuring Device (BPMD)

**Figure 10:**
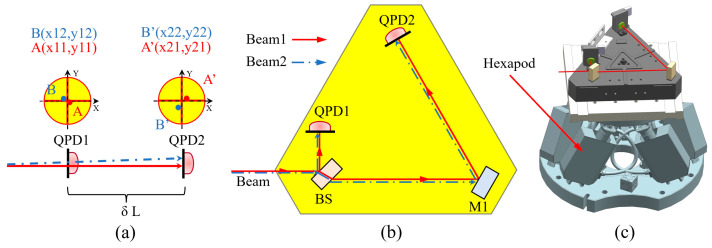
Measurement principle and model of BPMD. (**a**) The degree of coincidence can be measured by placing the QPD at different locations in the path of the two interference beams. (**b**) The use of BS and M1 can increase lever length and improve resolution. (**c**) The model when BPMD is used.

To accurately monitor the position of optical elements and beams during the installation and adjustment of the OBI, we developed a BPMD. As shown in Figure 10a, the BPMD uses the optical lever principle to measure the beam position and angular deviation. Figure 10b presents a schematic diagram of the optical path of the BPMD. The laser beam is evenly split at BS, one beam reaches QPD1, and the other beam continues to transmit on the OB, and it is detected at QPD2. QPD1 and QPD2 will measure the spot center of the laser beam. The Angle θ in the horizontal direction (parallel to the plane of the substrate) is given by Formula (4), and the Angle φ in the vertical direction (perpendicular to the plane of the substrate) is given by Formula (5). The distance between the two beams at a specific location (such as the detector receiving surface on the OBI) can be obtained through linear interpolation.

(4) $$\theta =\frac{\left(x_{22}-x_{21}\right)-(x_{12}-x_{11})}{\delta L}$$

(5) $$\varphi =\frac{\left(y_{22}-y_{21}\right)-(y_{12}-y_{11})}{\delta L}$$

As shown in Figure 10b,c, the base plate of the BPMD is triangular and machined from Invar. The structure of the base plate is optimized to improve the stiffness, reduce the mass, and reduce the influence of deformation on the measurement accuracy. BS and M1 are fabricated from fused silica and bonded to the Invar substrate using UV adhesive. When measuring the position and angular deviation of the two interference beams, the BPMD is placed on a hexapod (from Physik Instrumente, type: H-825.G2A, load 30 kg) capable of precise motion. The position and angle repeated accuracy of the hexapod are better than 0.5 μm and 2.5 μrad. The position measurement resolution of the QPD (QP50-6SD2 from First Sensor) is δx = 1 μm, δL = 280 mm, and the theoretical angular resolution of the BPMD is δx/δL = 3.6 μrad, which is better than 10 μrad in practice.

### 3.4. OBI Installation and Adjustment

Some of the Invar circular connection blocks are pre-installed on the Zerodur^®^ baseplate. The Invar is 4J32, which has a CTE of 1.5 × 10^−6^/K, which is close to the Zerodur^®^ baseplate, to avoid the effects of internal stress caused by temperature changes. Each circular connection block is machined with 4 threaded connection holes of 3 mm diameter, which are the interfaces for other no-HCB elements on the OBI to be fixed on the baseplate. The Invar blocks are bonded with the Zerodur^®^ baseplate using epoxy resin, with 19 on the A-side and 9 on the B-side.

Two FIOSs are mounted on the Zerodur^®^ baseplate; FIOSs are the light sources for the OBI, and they were purchased from Oz Optics Company, type: LPC-04-1060-6/125-P-3.5-18AS-40-3A-1-1. They have a large light spot with a diameter of 3.5 mm and a collimation distance of more than 3 m. They have been fitted with Invar fixtures and adjusted polarization before being mounted on the substrate. Using Scanning Slit Optical Beam Profilers (Thorlabs, type: BP209-IR/M), the height difference is controlled within 10 μm, and the Angle deviation is controlled within 5 μrad on a marble platform with a length of 6 m.

**Figure 11:**
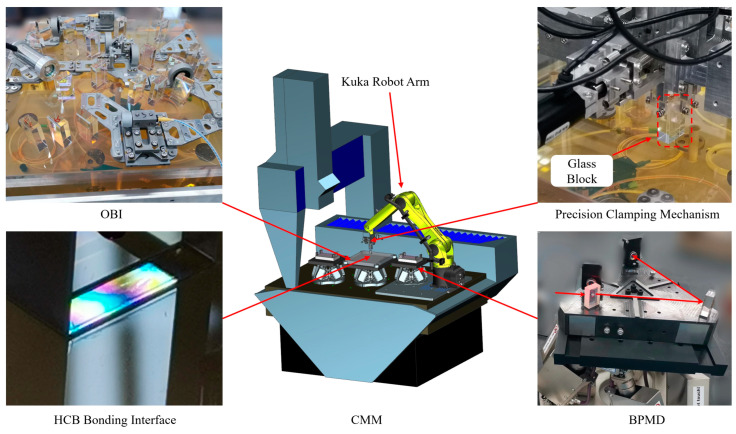
Schematic diagram of the OBI HCB. The OBI is placed on the CMM platform using two BPMDS to measure the beam position from different directions. The Kuka robotic arm and precision clamping machine are responsible for transporting and adjusting the glass block. A hexapod is used to place and adjust the OBI, and two hexapods are used to place the BPMDs. The CMM is the measuring tool.

The HCB is an important step. Figure 11 shows the schematic diagram of the HCB of the OBI. The bonding work was carried out on a marble platform with Coordinate Measuring Machines (CMMs) (type: ZEISS PRISMO ultra) in the laboratory with a cleanliness of thousands and an ambient temperature of 20 ± 0.2 °C. The bottom of the CMM is an air-floating platform, which insulates the influence of external vibration on the HCB. The measurement accuracy of the CMM is 0.5 + L/500 μm, and its temperature compensation system can automatically compensate for the impact of ambient temperature changes on the measurement. The BPMD in Section 3.3 can ensure that the beam position on the OBI is accurately measured.

The complete HCB steps are as follows:

1. The Zerodur^®^ baseplate is placed on the CMM, and under it is a hexapod, which raises and levels the OBI to facilitate subsequent measurement of the beam position by the BPMD. The hexapod can load 30 kg, and it has a power-off hold function, so the power can be cut off after adjusting the substrate position within a certain range.
2. Place the template for the initial positioning of the glass block on the Zerodur^®^ baseplate. Use the CMM to measure the position of the template and the position of each glass block to ensure that the position deviation is less than 5 μm. The glass blocks (such as BSa1, PBS1, PBS3, etc.) on the OBI with little influence on the optical path and low sensitivity were bonded through hydroxide catalysis, and 0.8 μL sodium silicate solution was used for each glass block.
3. For optical components with a large influence on the optical path and high sensitivity (such as BS3, BS5, BS6, etc.), the positioning accuracy of the template is not enough, and it is necessary to use the precision clamping mechanism to assist the installation and adjustment. Using the mechanical arm to move the glass block within 2 mm of the intended bonding position and at a height of approximately 3 mm from the Zerodur^®^ baseplate, the glass is pressed down on the Zerodur^®^ baseplate by finger 4, located at the top of the glass block (Figure 9), so that the bottom surface of the glass block is contacted to the surface of the Zerodur^®^ baseplate. Finger 4 is then pushed back (about 1 μm away). Adjust fingers 1, 2, and 3 for small movement and rotation of the glass block on the Zerodur^®^ baseplate. After adjusting the position of the glass block to the specified position, use the mechanical arm to lift the whole glass block, use the pipette gun to drop 0.8 μL solution on the Zerodur^®^ baseplate, and then operate the mechanical arm to reset down to the position 2 mm away from the substrate. At this time, the position of the glass block has a small deviation from the ideal position, which is determined by the reset accuracy of the mechanical arm. Use fingers 1, 2, and 3 to re-adjust the glass block to the specified position, and, finally, use finger 4 to press the glass block down to make full contact with the substrate and hold the solution between the two. After maintaining this state for 24 h, the bond already has a higher strength. Then, slowly back the four fingers more than 1 cm apart; because the clamping force is very small (only about 1.2 N), this will not destroy the bonding. Then, slowly move the mechanical arm away from the glass block completely to complete the bonding.

**Figure 12:**
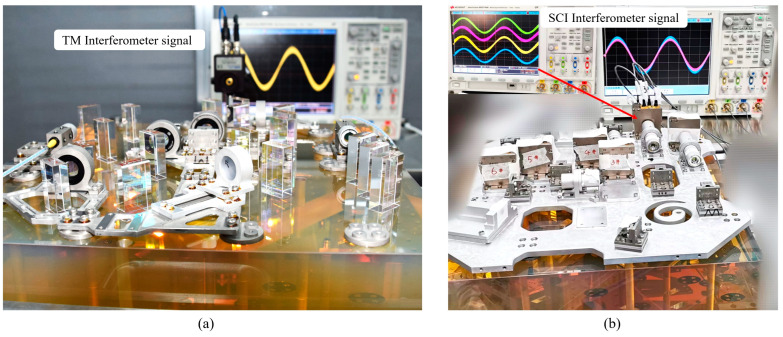
OBI construction diagram. (**a**) TM interferometer signals are monitored in real time when HCB is performed on the A-side. (**b**) SCI interferometer signals are monitored in real time when QPD is installed on the B-side. Numbers 1 and 2 (not shown) represent the QPDs of the scientific interferometer, numbers 3 and 4 represent the QPDs of the TM interferometer, and numbers 5 and 6 represent the QPDs of the reference interferometer.

The whole HCB process uses the BPMD and the CMM to measure the position of the beam and the QPD to measure the interference signal strength of the interference optical path in real time, as shown in Figure 12a. After completing the HCB of the A-side optical element, the coincidence degree of the three interference optical paths near the predetermined detector position was measured using BPMD, and the coincidence degree of the three interference optical paths reached 30 μm and 50 μrad (limited by the installation accuracy and processing accuracy).

After completing the HCB, the rest of the work becomes much easier. The HWPs and QWPs on the OBI are purchased from Thorlabs, and the small angle shift of the wave plates does not change the direction of the beam propagation; they can be bolted to the baseplate. The periscopes are also bolted to the baseplate at an Angle of 45 degrees from the plane of the baseplate. Before being fixed to the baseplate, they are adjusted using a theodolite as a measuring tool to ensure that the Angle deviation is less than 10 μrad.

There are no HCB elements on the B-side. The first step is to fix a metal plate to the baseplate through the reserved Invar interface. The stiffness of the metal plate is very good, and it will not cause deformation. The second step is to fix the periscopes in the predetermined position. The third step is to fix the BSMGs. The BSMG reduces the spot diameter from 3.5 mm to 1 mm, and the BSMGs for TM interferometers and scientific interferometers also have the function of partially suppressing the beam TTL jitter. The entry pupil of the TM interferometer BSMG is designed at the surface of the TM-reflected laser, and the exit pupil is designed at the QPD receiving laser plane, which ensures that when the TM deflects around the reflection center at a small Angle, the laser can still reach the QPD center after passing the mirror group. The entry pupil of the scientific interferometer BSMG is designed at the exit pupil of the telescope receiving laser, and the exit pupil is at the QPD receiving laser plane, which can also partially suppress the effect of the telescope TTL jitter. The last step is to install the QPD, which needs to ensure that the beam reaches the center of the detector and that the amplitudes and phases of the interference signal in the four quadrants of the QPD are consistent. As shown in Figure 12b, the oscilloscope shows that the amplitudes and phases of the four quadrants of the QPD are consistent.

**Figure 13:**
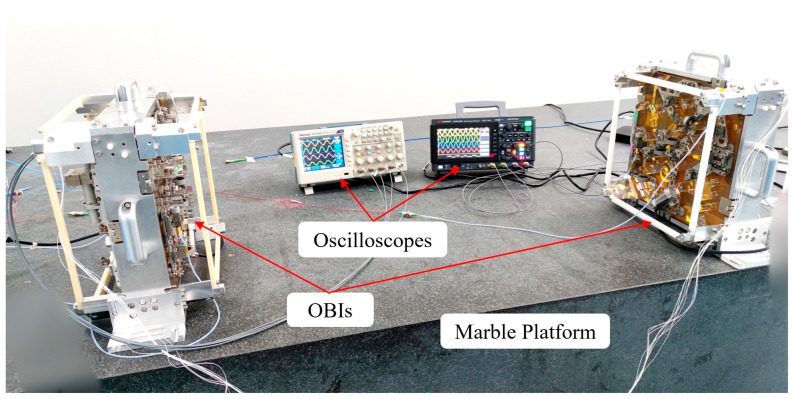
Two completed OBIs were placed on a marble platform. After the lasers are introduced into the OBIs, the obvious and stable interference signals are obtained, indicating that the HCB is firm and reliable.

Figure 13 shows two completed OBIs placed on a laboratory marble platform and waiting to enter the vacuum tank for experiments.

## 4. Stability Testing

**Figure 14:**
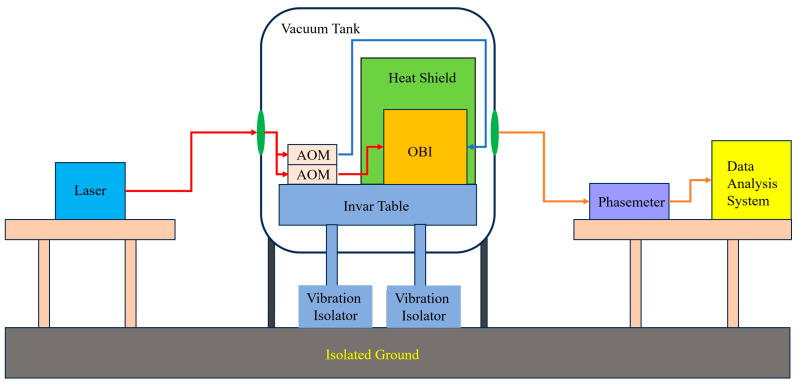
Block diagram of test system structure. The AOMs and OBI are placed inside the vacuum tank, and the laser light source, phase meter, and data analysis system are placed outside the vacuum tank for laser and data transmission through the flange interface on the vacuum tank. The Invar table reduces the impact of ambient vibration on the OBI, and the heat shield reduces the impact of ambient temperature fluctuations on the OBI.

After the OBIs were built, they were first placed in a constant temperature and humidity laboratory environment for a month to maximize the strength of the HCB. Experiments were carried out to evaluate the optical path-length stability of the OBI. The reference interferometer and the TM interferometer signals of a single OBI were collected, and the stability of the OBI was evaluated by comparing the reference interferometer and TM interferometer signals. In the TM interferometer, a mirror glued to the B-side of the baseplate was used instead of the TM. The experimental system structure block diagram is shown in Figure 14. The laser light source and signal acquisition equipment are placed outside the vacuum tank, and the OBI and AOMs are placed on the Invar table inside the tank.

**Figure 15:**
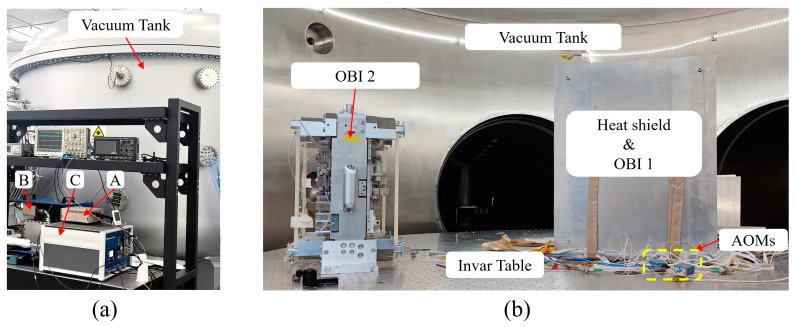
Test site. (**a**) Outside the vacuum tank frequency-stabilized laser system, where A is the seed light source, B is the frequency-stabilized cavity, and C is the control electronics box. (**b**) Inside the vacuum tank; two OBIs and AOMs are placed on the Invar table, and OBI1 is insulated for the experiment. The “OBI 2” was only placed in the vacuum tank and did not participate in the test.

As shown in Figure 15a, the Laser light sources are placed outside the vacuum tank; A is the seed light source, B is the frequency stabilizer cavity, and C is the control electronics box. The stable laser system type is SLS-1064-300-1000, with an output power of 145 mW and a wavelength of 1064 nm. The frequency stability of the laser can reach 550 Hz every three hours, and the power stability is 99.96%. The laser is introduced into the vacuum tank through the fiber flange, and it is split into two beams in the vacuum tank. The beams are modulated by AOMs (type: SGTF150-1064-1P) into the center frequency of 130 MHz and 131.6 MHz with a difference frequency of 1.6 MHz and then imported into the OBI by FIOSs. The vacuum tank is 3 m in diameter, and it contains a 2 m diameter Invar table, which is vibration isolated. The vacuum degree of the vacuum tank is 0.25 Pa, and the OBI is insulated in a heat shield to reduce the influence of external thermal conduction and thermal radiation on it.

A 16-channel phase meter was used to measure the optical information of four QPDs of the OBI reference interferometer and TM interferometer. The interference signal was converted into an electrical signal using a 14-bit digital-to-analog converter. The sampling frequency of the phase meter was 80 MHz. The phase change information of each channel is extracted through 16 digital PLLs, and the output rate of the phase information is 20 Hz.

**Figure 16:**
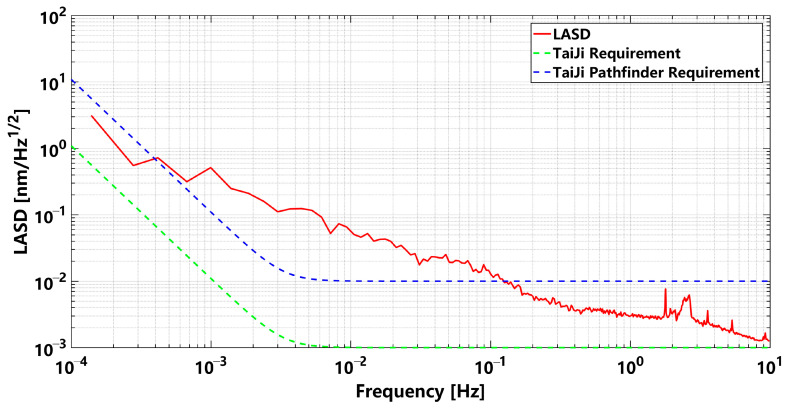
Noise spectrum of OBI stability test results. The green dotted line is the Taiji’s noise requirement for the OBI, the blue dotted line is the Taiji Pathfinder’s noise requirement for the OBI, and the red solid line is the noise curve of the OBI we constructed.

The test lasted for 96.8 h. As shown in Figure 16, the data of a period (73~75 h) were selected to make a spectrum, and the OBI noise spectral density diagram was obtained. The noise spectral density was calculated using the LTPDA toolbox developed by the Albert Einstein Institute (Hannover, Germany) [19]. In the figure, the red curve is the actual OBI stability curve, and the blue dashed line and the green dashed line are the noise requirement curves of the Taiji Pathfinder and Taiji mission, respectively. It can be seen that the OBI noise is less than 10 pm/√Hz in the band range of 0.1 Hz to 1 Hz, and it reaches 3 pm/√Hz at 1 Hz, which has met the requirements of the Taiji Pathfinder in the middle- and high-frequency band. The OBI noise is less than 100 pm/√Hz in the band from 3 mHz to 0.1 Hz. Excessive noise below 0.1 Hz may come from laser frequency noise, ambient temperature drift noise, laser pointing jitter coupled with optical path-length noise caused by FIOS, electronics noise, and OBI stray light noise. In the follow-up study, we will find the sources of OBI noise and its suppression methods.

## 5. Conclusions and Outlook

Two full-function two-side OBIs were designed and constructed by using the low-stress bonding technique of HCB. The precision gripper auxiliary setting mechanism and BPMD were developed, and they realized the corresponding functions in the construction of the OBI. The three interference optical paths on the constructed OBI have a high coincidence degree, and the position and angle deviations are less than 30 μm and 50 μrad. The stability of the OBI was tested, and the test results show that when the frequency is greater than 0.1 Hz, the OBI noise is less than 10 pm/√Hz, which meets the requirements of the Taiji Pathfinder in the middle- and high-frequency band.

It is worth noting that the noise below 0.1 Hz is still too high on the OBI. At the same time, the laser source FIOS, periscope, and BSMG are not all glass, and they are not bonded to the baseplate using HCB, so they will still bring some noise to the OBI. Research on an all-glass FIOS has made some progress, and we will carry out further research on an all-glass periscope, an all-glass BSMG, OBI stray light suppression, and other noise suppression to meet the final requirements of the Taiji task.

## Figures and Tables

**Table 1:** Description of each optical component of the OBI (single).

Abbreviation	Full Name	Number
M	Mirror ^1^	5
BS	Beam Splitter (R:T is 50:50)	6
BSa	Beam Splitter (R:T is 10:90)	1
BSb	Beam Splitter (R:T is 90:10)	1
BSc	Beam Splitter (R:T is 1:99)	1
BSd	Beam Splitter (R:T is 99:1)	2
PBS	Polarizing Beam Splitter	3
FIOS	Fiber Injector Optical Subassembly	2
P-A	Periscope ^2^ in side A	9
P-B	Periscope in side B	9
HWP	Half Wave Plate	2
QWP	Quarter Wave Plate	2
Telescope-I/F	Telescope Interface	1
TM-I/F	Test Mass Interface	1
PAAM	Point Ahead Angle Mechanism	1
BSMG	Beam Shrinking Mirror Group	7
QPD	Quadrant Photodiode	7

^1^ The Mirror is plated with a full reflective dielectric film. ^2^ The periscope is a gold-plated mirror with a diameter of 7 mm.

## Data Availability

No new data were created or analyzed in this study.

## References

[1] Abbott B., Abbott R., Abbott T.D., Abernathy M.R., Acernese F., Ackley K., Adams C., Adams T., Addesso P., Adhikari R.X. (2016). LIGO Scientific Collaboration and Virgo Collaboration (2016) Directly comparing GW150914 with numerical solutions of Einstein’s equations for binary black hole coalescence. Phys. Rev. D.

[2] Collaboration L.S., Virgo C., Abbott B.P., Abbott R., Abbott T.D., Abernathy M.R., Acernese F., Ackley K., Adams C., Adams T. (2016). Observation of Gravitational Waves from a Binary Black Hole Merger. Phys. Rev. Lett..

[3] Luo Z.R., Wang Y., Wu Y.L., Hu W.R., Jin G. (2021). The Taiji program: A concise overview. Prog. Theor. Exp. Phys..

[4] Xia Y., Li G.Y., Heinzel G., Rüdiger A., Luo Y.J. (2010). Orbit design for the Laser Interferometer Space Antenna (LISA). Sci. China-Phys. Mech. Astron..

[5] Bayle J.B., Bonga B., Caprini C., Doneva D., Muratore M., Petiteau A., Rossi E., Shao L.J. (2022). Overview and progress on the Laser Interferometer Space Antenna mission. Nat. Astron..

[6] Armano M., Audley H., Baird J., Binetruy P., Born M., Bortoluzzi D., Brandt N., Castelli E., Cavalleri A., Cesarini A. (2022). Sensor noise in LISA Pathfinder: An extensive in-flight review of the angular and longitudinal interferometric measurement system. Phys. Rev. D.

[7] Gao R.H., Liu H.S., Zhao Y., Luo Z.R., Jin G. (2021). Automatic, high-speed, high-precision acquisition scheme with QPD for the Taiji program. Opt. Express.

[8] Beveridge N.L. (2012). Characterisation of Silicon-Silicon Hydroxide Catalysis Bonds for Future Gravitational Wave Detectors. Ph.D. Thesis.

[9] Robertson D.I., Fitzsimons E.D., Killow C.J., Perreur-Lloyd M., Ward H., Bryant J., Cruise A.M., Dixon G., Hoyland D., Smith D. (2013). Construction and testing of the optical bench for LISA Pathfinder. Class. Quantum Gravity.

[10] Armano M., Audley H., Auger G., Baird J.T., Bassan M., Binetruy P., Born M., Bortoluzzi D., Brandt N., Caleno M. (2016). Sub-Femto-g Free Fall for Space-Based Gravitational Wave Observatories: LISA Pathfinder Results. Phys. Rev. Lett..

[11] Armano M., Audley H., Baird J., Binetruy P., Born M., Bortoluzzi D., Castelli E., Cavalleri A., Cesarini A., Cruise A.M. (2018). Beyond the Required LISA Free-Fall Performance: New LISA Pathfinder Results down to 20 μHz. Phys. Rev. Lett..

[12] Preston A., Thorpe J.I., Miner L., IEEE Quasi-monolithic structures for spaceflight using hydroxide-catalysis bonding. Proceedings of the IEEE Aerospace Conference.

[13] Vitale S. (2014). Space-borne gravitational wave observatories. Gen. Relativ. Gravit..

[14] Wang S.W., Lipa J.A., Gwo D.H., Triebes K., Turneaure J.P., Farley R.P., Davidson D., Bower K.A., Acworth E.B., Bernier R.J. (2015). The design and performance of the Gravity Probe B telescope. Class. Quantum Gravity.

[15] Chwalla M., Danzmann K., Alvarez M.D., Delgado J.J.E., Barranco G.F., Fitzsimons E., Gerberding O., Heinzel G., Killow C.J., Lieser M. (2020). Optical Suppression of Tilt-to-Length Coupling in the LISA Long-Arm Interferometer. Phys. Rev. Appl..

[16] Chwalla M., Danzmann K., Fernández Barranco G., Fitzsimons E., Gerberding O., Heinzel G., Killow C.J., Lieser M., Perreur-Lloyd M., Robertson D.I. (2016). Design and construction of an optical test bed for LISA imaging systems and tilt-to-length coupling. Class. Quantum Gravity.

[17] van Veggel A.M.A., Killow C.J. (2014). Hydroxide catalysis bonding for astronomical instruments. Adv. Opt. Technol..

[18] Elliffe E.J., Bogenstahl J., Deshpande A., Hough J., Killow C., Reid S., Robertson D., Rowan S., Ward H., Cagnoli G. (2005). Hydroxide-catalysis bonding for stable optical systems for space. Class. Quantum Gravity.

[19] Heinzel G., Wand V., García A., Jennrich O.P., Braxmaier C., Robertson D., Middleton K., Hoyland D., Rüdiger A., Schilling R. (2004). The LTP interferometer and phasemeter. Class. Quantum Gravity.

